# Effects of Combined Interventions of Exercise and Diet or Exercise and Supplementation on Breast Cancer Patients: A Systematic Review

**DOI:** 10.3390/nu15041013

**Published:** 2023-02-17

**Authors:** Txomin Pérez-Bilbao, María Alonso-Dueñas, Ana B. Peinado, Alejandro F. San Juan

**Affiliations:** 1 Department of Health and Human Performance, Faculty of Physical Activity and Sports Sciences (INEF), Universidad Politécnica de Madrid, 28040 Madrid, Spain; 2 GEICAM Spanish Breast Cancer Group, 28703 Madrid, Spain; 3 LFE Research Group, Universidad Politécnica de Madrid, 28040 Madrid, Spain

**Keywords:** breast cancer, diet, supplementation, physical activity, exercise, quality of life, body composition, physical function, healthy lifestyle, psychological variables, fatigue

## Abstract

This systematic review investigated the effects of exercise interventions combined with diet and/or dietary supplement interventions on anthropometry, body composition, metabolic biomarkers, physical function, healthy lifestyles, quality of life, psychosocial variables and fatigue for women with breast cancer. A systematic search was performed in the PubMed and Web of Science databases (from inception to 1 March 2022). A review was carried out following the Preferred Reporting Items for Systematic review and Meta-Analyses (PRISMA) guidelines. The methodological quality and the risk of bias of the included studies was assessed with the Physiotherapy Evidence Database (PEDro) scale. A total of 13 randomised controlled trial studies were included, comprising 1569 breast cancer patients. The main finding of this systematic review is that groups performing interventions combining exercise plus diet show significant improvements in cardiorespiratory fitness, muscular strength, body composition, quality of life, fatigue, anxiety, depression and sleep compared to control groups. On the other hand, the use of interventions combining exercise plus supplementation does not result in an improvement compared to groups using exercise alone or supplementation alone.

## 1. Introduction

Breast cancer (BC) was the most diagnosed cancer in 2020, with an estimated 2.26 million new cases, representing 12.5% of all cancer cases in the world [1]. It had been estimated that in 2022 the number of women diagnosed with BC in Spain would be 34.750 [1]. However, the 2022 reality may be slightly different, as this estimate does not include the possible effect of the COVID-19 pandemic [1].

Physical activity (PA), diet and weight management play a major role among BC patients. The numerous benefits of PA in prevention during treatment and survival have been well documented. In 2018 the Physical Activity Guidelines Advisory Committee review concluded that there is strong evidence for the influence of PA on decreasing risk in seven types of cancer including BC [2]. Moreover, exercise, and specifically aerobic and resistance exercise, is shown to have positive influence on cardiopulmonary function and several biomarkers (i.e., insulin, leptin, Tumour Necrosis Factor alpha (TNF-α) or sex hormone binding globulin), bone loss, body composition, depression and anxiety, and quality of life (QoL), among other factors, during BC treatment [3,4,5]. Although the real impact of PA on the risk of relapse and cancer mortality is not well defined, the evidence can conclude that PA can contribute to reducing cancer-related mortality and all-cause mortality in cancer survivors [6].

According to the international dietary guidelines, a healthy dietary pattern includes a high consumption of fruits, a variety of vegetables, whole grains, poultry and fish and a low consumption of red meat, alcohol, sugar-sweetened beverages, highly processed foods and refined grain products [7]. These recommendations are similar to the Mediterranean diet which includes, in addition to the above foods, extra-virgin olive oil, nuts, legumes, eggs and dairy [8]. The potential of nutrition in BC survivors has been increasingly studied over the years, but with inconsistent results [9,10]. Moreover, association between dietary factors and BC outcomes has long been studied in several cohort studies, founding a lower risk of death from non-breast cancer causes with healthy dietary pattern [11,12,13,14]. In a meta-analysis of cohort studies carried out by Hou et al. [15] concluded that women diagnosed with breast cancer may benefit from healthy dietary patterns and improve their survival rates.

Further, dietary supplements are commonly used in BC survivors, more than in other cancer survivors [16,17]. Overall, supplement intake is based on antioxidants and multivitamins such as vitamins C, D and E [18]. However, these products may interact positively or negatively with cancer-related treatments, so their safety, efficacy, timing and dose should be taken with caution [19]. Thus, international recommendations indicate that dietary supplementation should only be considered when it is advised by a trained health professional and there is a nutritional deficiency [20,21].

Due to the limited number of interventions accurately describing combined exercise and diet and/or supplementation in BC patients, we conducted a systematic review to provide an overview of the current evidence on interventions that combine exercise and diet and/or supplementation in BC patients.

## 2. Materials and Methods

A systematic literature review was conducted based on the Preferred Reporting Items for Systematic Reviews and Meta-Analyses (PRISMA) guidelines [22].

### 2.1. Search Strategy

An electronic search of PubMed and Web of Science was conducted from inception to 1 March 2022. We used the Mesh indexed terms from PubMed and the following search strategy: (breast OR mammary) AND (cancer or neoplas* OR malignant OR carcinoma OR tumour OR adenoma OR polyps) AND (“physical activity” or “physical exercise” OR training OR exercise) AND (diet* OR nutrition* OR “calorie restrict*” OR “caloric restrict*” OR “weight loss” OR “weight intervention” OR “dietary supplement*” OR “nutritional supplement*” OR “supplement*” OR “omega*” OR “calcium” OR “vitamin*” OR “creatine” OR “glutamine” OR “mineral*” OR “antioxidant*” OR “amino acid*” OR “soy” OR “whey protein”).

### 2.2. Elegibility Criteria

The inclusion criteria were specified by the Population, Intervention, Control, Outcomes, Study design (PICOS) framework. These included (1) population: women with a histologically confirmed diagnosis of breast cancer (including all stages of, and treatments for, breast cancer); (2) intervention: any detailed and accurate combined exercise and diet or exercise and supplementation protocols, for any duration; (3) control: comparison group receiving a combined exercise and diet and supplementation protocol of a lesser intensity, control group (CG) not receiving the intervention at any time point during the trial or waiting list control care; (4) outcomes: changes in health-related variables (e.g., physical condition, blood analysis, nutritional status), QoL and psychosocial variables; and (5) study design: randomised control trials (RCTs) with a Physiotherapy Evidence Database (PEDro) score ≥ 6. Only full-text English articles of human trials, published in peer-reviewed journals were included in the search process.

### 2.3. Study Selection and Data Extraction

In a first step, any duplicates were manually removed. Then, the titles and abstracts of articles were checked for relevance by two researchers (T.P.B. and M.A.). They subsequently, independently from each other, reviewed the full texts of potentially eligible articles. Any disagreements were discussed with a third researcher (A.F.S.J.) until consensus was reached.

The authors, publication date, number of patients, age of patients, cancer stage, cancer treatment, dietary supplement(s) and dosing regime, details of dietary intervention, information on the exercise intervention (training type, training frequency, training intensity, supervision, setting), duration of the program, list of outcome variables and main statistical results (changes from baseline within and/or between groups) were extracted from the studies by two authors (T.P.B. and M.A.) independently. If there were disagreements in data extraction, authors discussed until a consensus was reached.

### 2.4. Quality Assessment and Risk of Bias

The Physiotherapy Evidence Database (PEDro) scale was used for the assessment of the risk of bias among the included studies. The PEDro scale is a valid measure of the methodological quality of the clinical trial [23]. Two authors (T.P.B. and M.A.) independently scored the studies and a third author (A.F.S.J.) was recruited in cases of any argument. The PEDro scale consists of 11 items including external validity (item 1), internal validity (items 2–9) and statistical reporting (items 10 and 11) and each one of them contributes 1 point to a total score of 10, excluding item 1. The following cut-points were used to categorise studies by quality: excellent (9–10); good (6–8); fair (4–5); poor (<4) [24].

## 3. Results

### 3.1. Study Selection

Overall, 1090 records were identified through all database searches. After 171 duplicates had been removed, titles and abstracts from the remaining 919 records were screened and the full text of 568 was assessed for eligibility. Finally, 13 studies were included in this systematic review describing 12 interventions [25,26,27,28,29,30,31,32,33,34,35,36,37] (i.e., one intervention published 2 studies showing its results). We were forced to discard one study conducted by Arikawa et al. [38] because the p-values were not reported, even though some variable changes, body mass and fitness level, were listed as significant in the body of the article. After writing to the authors for p-values and receiving no response, we did not include the results of this study in this systematic review, but they will be mentioned in the corresponding sections of the discussion.

**Figure 1:**
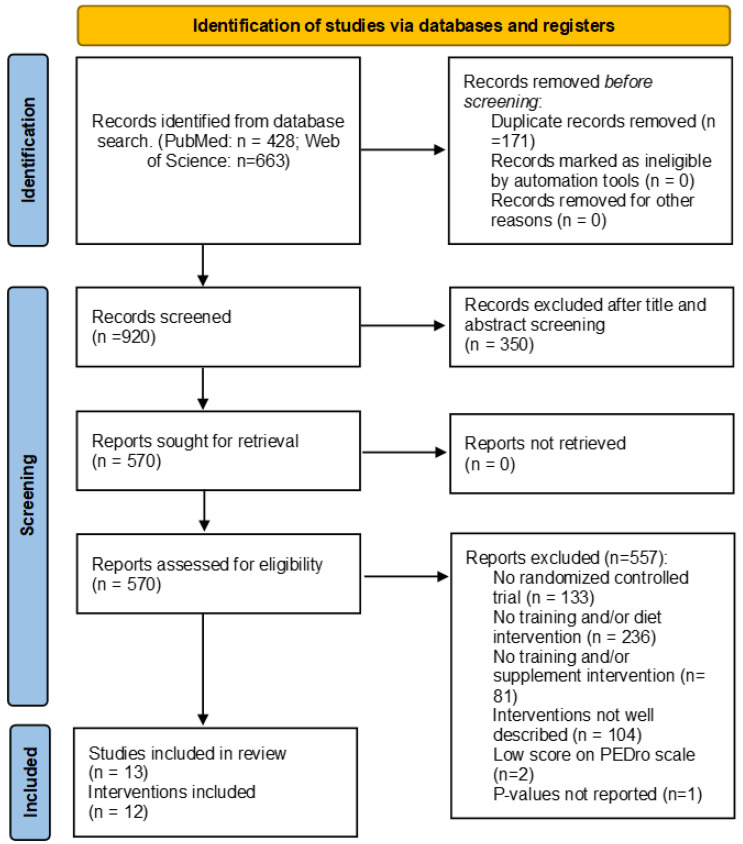
PRISMA flow diagram for the Systematic Review.

A comprehensive flow diagram of the study selection process is presented in Figure 1.

### 3.2. Study Characteristics

Characteristics of the included studies are presented in Table 1 and Table 2.

#### 3.2.1. Participants

A total of 1569 breast cancer patients were included across the 12 interventions. Sample sizes ranged from 25 [25] to 409 [28] patients. The average age of patients ranged from 49.0 [30,31] to 58.7 years [37]. Eight interventions included patients receiving exercise plus diet interventions [26,27,28,30,31,33,34,35,36], and four interventions included patients receiving exercise plus supplementation interventions [25,29,32,37]. Of the 12 interventions analysed, in 11 of them, participants had a body mass index (BMI) > 25 kg/m^2^ [25,26,27,28,29,30,31,33,34,35,36,37]. In the other intervention, the BMI of the CG was 23.4 kg/m^2^ and of the experimental group (EG) was 23.3 kg/m^2^ [32]. Regarding menopausal status, 4 interventions did not report it [25,30,31,33,34], in 2 interventions all participants were postmenopausal [32,37] and in 6 interventions, postmenopausal participants were between 40.0% and 90.0% of the total participants [26,27,28,29,35,36]. Regarding hormone therapy (HT) intake by participants, in 6 interventions they were receiving tamoxifen and aromatase inhibitors [28,29,34,35,36,37], 1 intervention tamoxifen [30,31], 1 intervention aromatase inhibitors plus selective estrogen receptor modulator [32], in 1 intervention the type of HT was not specified [25] and in 3 interventions it was not specified whether they were on HT or not [26,27,33]. Among all interventions, there were two interventions where all participants were receiving HT [29,30,31].

#### 3.2.2. Exercise Interventions

Supervision: Exercise programs were supervised in 4 interventions (33%) [25,30,31,34,36], semi-supervised in 5 interventions (42%) [26,27,28,33,37], 2 interventions were not supervised (17%) [29,32] and 1 intervention did not report supervision (8%) [35].

Frequency: A total of 2 interventions (17%) performed exercise programs > 5 times per week [28,29]; 7 interventions (58%) performed exercise programs 3–5 times per week [27,30,31,32,33,34,35,36]; and 3 interventions (25%) exercised twice per week [25,26,37].

Intensity training: Seven interventions (58%) offered no information regarding strength training [26,27,28,32,34,36,37]. In 3 interventions (25%), the intensity exercise was evaluated using a perceived exertion scale [29,32,33], 5 interventions (42%) reported aerobic intensity exercise using a percentage of maximal or peak heart rate [28,33,34,35,36], 4 interventions (33%) reported aerobic intensity exercise using a percentage of heart rate reserve [26,27,30,31] and 1 intervention (8%) reported strength training intensity using the method of maximum repetitions (RM) [25].

Duration: A total of 4 interventions (33%) lasted up to 12 weeks [25,28,29,33], 5 interventions (42%) lasted between 13 and 24 weeks [30,31,32,34,35,36] and 3 interventions (25%) lasted more than 24 weeks [26,27,37]. The most common duration was 24 weeks, with 5 interventions (42%) being performed for this duration. The strength training duration per session lasted 10 to 15 min in 1 intervention [36], 30 to 45 min in another intervention [37] and was not reported in 6 interventions [25,26,27,28,32,34]. Aerobic training lasted 30 min in 4 interventions [28,34,35,36], 20 to 60 min in 1 intervention [32] and 50 to 65 min in 2 interventions [26,27]. One intervention did not adequately report the duration of the exercise sessions [29].

Type: Most of the studies included multicomponent exercise interventions. Seven interventions (58%) focused on strength and aerobic training [26,27,28,29,32,34,36], three interventions (25%) included only aerobic training [30,31,33,35], one intervention (8%) included strength and balance [37] and one intervention (8%) included strength training only [25]. When reported, the most used activities for aerobic training were walking and cycling. For strength training, the most common resistances used were elastic bands. For a more detailed description, please see Table 1 and Table 2.

Delivery setting: Four interventions (33%) were delivered both at the participant’s homes and at exercise facilities [28,30,31,33,37], three interventions (25%) at exercise facilities [34,35,36], two interventions (17%) were delivered both in the participant’s homes and in health care settings [26,27], two interventions (17%) at the participant’s home [29,32] and one intervention (8%) did not report delivery setting [25].

#### 3.2.3. Diet Interventions

Of the 12 interventions included, 8 used a combined diet and exercise intervention (66.6%).

Type: Five interventions (62.5%) included a hypocaloric diet [28,30,31,34,35,36], two interventions (25%) included a normocaloric diet [26,27] and one intervention (12.5%) did not report the type of diet [33].

Macronutrient composition: Two interventions (25%) included a diet with a macronutrient composition of 30–35% lipids, 50–55% carbohydrates and 10–15% proteins [26,27], one intervention (12.5%) had a diet composition that was 30% lipids, 45% carbohydrates and 25% proteins [28], another intervention (12.5%) had a diet composition that was 25% lipids, 30% carbohydrates and 45% proteins [35] and four interventions (50%) did not report diet composition [30,31,33,34,37].

#### 3.2.4. Supplement Interventions

Of the 12 interventions included, 4 used a combined supplement and exercise intervention (33.3%).

Type: Two interventions (50%) included a calcium and vitamin D supplementation [32,37], one intervention (25%) included a vitamin D supplementation [29] and one intervention (25%) reported a supplementation with vitamin C and vitamin E [25].

#### 3.2.5. Compliance Rate

A total of 11 out of 12 interventions (92%) reported the rate of adherence of their participants to exercise, diet and supplementation. The compliance rate to exercise was <40% [35], 40–79% [26,27,28,32,37], 80–90% [33,34,36], >90% [25] and not specified [29,30,31]. The compliance rate to diet was <70% [28], 70–90% [33,35,36], >90% [27] and not specified [26,30,31,34]. Finally, the compliance rate to supplementation was 80–90% [32] and >90% [25,29,37].

### 3.3. Quality Assessments and Risk of Bias

The interventions included in this systematic review presented PEDro scores that ranged between 6 and 9 points with an average score of 6.9 points. Ten of them had good scores [26,27,28,29,30,31,33,34,35,36,37] and two had excellent scores [25,32]. The PEDro score of each included study is presented in Table 3.

### 3.4. Outcome Measures and Results of Included Studies

The results of the studies receiving exercise plus diet interventions and exercise plus supplementation interventions are presented in Table 4 and Table 5, respectively. In addition, a summary table has been added to facilitate the understanding of the results (Table 6).

#### 3.4.1. Anthropometry, Body Composition and Metabolic Biomarkers

Anthropometry and body composition was analysed in 7 of 12 interventions (58.3%) using BMI [26,27,30,31,33,36], body mass (BM) [26,27,28,33,35,36], waist circumference [26,28,33,35,36], hip circumference [28,33,35], fat mass (FM) [27,28,33,35], muscle mass [27], fat free mass (FFM) [28,35] and bone mineral density (BMD) [32,37]. In 6 of the 7 interventions (85.7%), significant improvements were observed in the following variables: BMI [27,31,33], BM [28,33,35], waist circumference [28,35,36], hip circumference [28,35], FM [27,28,33,35], muscle mass [27] and FFM [28].

The techniques used to measure FM were dual energy x-ray absorptiometry (DXA) [28,35], bioelectrical impedance analysis [27,36] and skinfold measurement [33]. In the case of muscle mass, bioelectrical impedance analysis was used [27] and finally for FFM, DXA was employed [28].

Metabolic biomarkers were analysed in 8 of 12 interventions (66.7%) using the following variables: total cholesterol (TC) [28,35,36], low-density lipoprotein cholesterol (LDL-C) [28,35], high-density lipoprotein cholesterol (HDL-C) [28,35,36], triglycerides (TG) [28,35], insulin [28,33,35], glucose [28,35], homeostatic model assessment of insulin resistance (HOMA-IR) [28,35,36], interleukin-6 (IL-6) [33,34], TNF-α [33,34], adiponectin [33], leptin [33,36], ghrelin [35], cortisol [34], leukocyte [34], insulin-like growth factor-I (IGF-I) [35,36], insulin-like growth factor binding protein-1 (IGFBP-1) [35,36], insulin-like growth factor binding protein-3 (IGFBP-3) [35,36], C-reactive protein (CRP) [35,36], testosterone [36], oestradiol [36], estrone [36], sex hormone-binding globulin [36], type I collagen linked N-telopeptide [29,32,37], serum calcium [32], vitamin D (serum 25(OH)D) [32], bone-specific alkaline phosphatase [29], bone remodeling index [29] and alkphase B [37]. In 4 of the 8 interventions (50%) significant changes were observed between intervention and control group. In the intervention performed by Saxton et al. [34], cortisol increased in the intervention group. Significant improvements were observed in the following variables: leptin [36], TC [28,36], LDL-C [28], TG [28], insulin [28], HOMA-IR [28] and bone-specific alkaline phosphatase [29]. In one intervention (13%), HDL-C remained unchanged in the intervention group, whereas it decreased in the control group [36].

#### 3.4.2. Physical Function

Physical function was analysed in 9 of the 12 interventions (75%) with the following variables: muscular strength [27,29,32,37], muscular endurance [26,27], muscular power [27], balance [37], cardiorespiratory fitness (CRF) [28,29,32,33,35,36] and blood pressure [28,36]. In 5 of the 9 interventions (55.6%) significant improvements were observed in the following variables: muscular endurance [26,27], muscular strength [37], CRF [28,36], diastolic blood pressure [36] and systolic blood pressure [28].

Isometric muscular strength was measured using handgrip test [29,32] and wall-squat test [32], isokinetic muscular strength was measured using velocity spectrum evaluation [37] and dynamic muscular strength was measured using Chair-Stand test [32] and 10 vertical jumps test [27]. One intervention also measured dynamic muscular strength using chest press and leg extensions exercises; however, the type of protocol used was not specified [29]. Muscular power was determined using the 10 vertical jumps test [27], and muscular endurance was measured using Sit-to-Stand test at 15 and 30 s [26,27]. CRF was analysed using the following tests: 12-min Walking Test (12MWT) [28], 8-min Walking Test (8MWT) [36] and 6-min Walking Test (6MWT) [32]. The following lung function variables were measured directly with a gas analyser: Peak Oxygen Uptake (VO_2peak_) [33] and Maximal Oxygen Uptake (VO_2max_) [35]. One intervention also reported VO_2max_ but did not specify how it was determined [29].

#### 3.4.3. Healthy Lifestyles

Healthy lifestyles were analysed in 8 of the 12 interventions (66.7%). The variables studied were amount of physical activity [26,27,28,32,33,35], dietary intake [26,27,35,36] and sleep quality [31]. In 8 of the 8 interventions (100%) significant improvements were observed in amount of physical activity [26,27,28,32,33,35], dietary intake [26,27,28,35,36] and sleep quality [31]. In one intervention, both the EG and the CG improved in the total amount of physical activity, but no significant differences were found between the two groups [32]. More specifically, with respect to the amount of physical activity, the results were as follows: an increase in moderate recreational activity or leisure time [26,27]; a decrease in inactive time and an increase in active time [33]; and an increase in the sport/exercise index [35]. Regarding dietary intake, in the different interventions, an increase in the consumption of fibre [26,35] and fruit/vegetables [35], and a decrease in the consumption of alcohol [26], animal protein [26], total fat [28,36], saturated fat [28,36] and energy intake [28,35] were found.

Dietary intake was evaluated using food records, the amount of physical activity was measured using a Global Physical Activity Questionnaire [26,27], the Godin Leisure-Time Exercise Questionnaire [32], the Habitual Activity Estimation Scale [33], the Kaiser Physical Activity Survey [35] and a pedometer [28]. On the other hand, sleep quality was measured using the Pittsburgh Sleep Quality Index [31].

#### 3.4.4. Quality of Life

Quality of Life was analysed in 6 of the 12 interventions (50%) and all were exercise plus diet interventions. In 5 of the previous 6 interventions (83.3%), a significant improvement in QoL was observed in the intervention group compared to a control group [27,28,30,31,33,36]. In more detail, improvements in the European Organization for the Research and Treatment of Cancer Quality of Life Questionnaires (EORTC QLQ-C30) were found in Global QoL [27,30], Physical Function [27,30], Role Function [27,30], Emotional Function [27,30], Cognitive Function [27,30], Social Function [27,30], Fatigue [30], Nausea and Vomiting [30], Dyspnoea [30], Appetite [30], Constipation [30], Diarrhoea [30] and Financial Difficulties [30]. With respect to the EORTC-BR23, improvements were found in the Body Image [31] and Future Perspective subscales [31]. However, Sexual Function and Sexual Enjoyment subscales decreased significantly in the intervention group [31]. Finally, with respect to the Functional Assessment of Cancer Therapy-Breast (FACT-B), improvements were found in the following items: Emotional Wellbeing Subscale [33], Breast Cancer Subscale [33,36] and FACT-B Total Score [33,36].

They measured QoL throughout the EORTC QLQ-C30 for general cancer patients [26,27,30], EORTC QLQ-BR23 for breast cancer patients [31] and the Functional Assessment of Cancer Therapy-Breast (FACT-B) [28,33,36] questionnaires.

#### 3.4.5. Psychosocial Variables

Psychosocial variables were analysed in 4 of the 12 interventions (25%). These exercise plus diet interventions measured the following variables: Depression [26,27,34], Anxiety [26,27], Stress [34] and Economic Evaluation [28]. In all the mentioned interventions (100%) significant improvements were observed in all the psychological variables studied.

Depression was measured using Hospital Anxiety Depression Scale [26,27] and the Beck Depression Inventory-II [34], Anxiety was measured using Hospital Anxiety Depression Scale [26,27] and Stress was measured using the Perceived Stress Scale [34]. Regarding the Economic Evaluation, both the home group (£7737) and the community group (£7914) had reduced patient costs with respect to the control group (£8547). This is largely due to the reduction in medication to treat toxicity, fewer physiotherapy visits and fewer accidents and emergency visits [28].

#### 3.4.6. Fatigue

Fatigue was analysed in 4 of the 12 interventions (25%). In 2 of 4 interventions (50%) a significant improvement in Fatigue was observed [27,31]. The interventions that measured fatigue using the MFI showed improvements in the following items: General Fatigue [27], Physical Fatigue [27], Mental Fatigue [27], Reduced Activities [27] and Reduced Motivation [27]. On the other hand, the intervention that measured Fatigue using CFS showed a significant improvement in the Fatigue overall score [31].

Fatigue was measured throughout the Multidimensional Fatigue Inventory (MFI) [25,26,27] and Cancer Fatigue Scale (CFS) [31].

## 4. Discussion

This systematic review investigates the effects of exercise interventions combined with diet and/or dietary supplement interventions on Anthropometry, Body Composition and Metabolic Biomarkers, Physical Function, Healthy Lifestyles, QoL, Psychosocial Variables and Fatigue on women with breast cancer. The main finding of this systematic review is that groups performing interventions combining exercise plus diet show significant improvements in CRF, muscular strength, body composition, QoL, fatigue, anxiety, depression and sleep compared to control groups. On the other hand, the use of interventions combining exercise plus supplementation does not result in an improvement compared to groups using exercise alone or supplementation alone.

We have not found any systematic review that has analysed the effects of a combined intervention of exercise and/or dietary supplementation in breast cancer patients. To the best of our knowledge, there are two systematic reviews with meta-analysis [39,40] and one systematic review [41] that studied the effects of exercise in combination with a nutritional intervention in breast cancer patients on weight loss and biomarkers of inflammation. Our systematic review presents similarities with these three papers: (1) exercise interventions varied greatly, (2) combined diet plus exercise interventions showed improvements in the variables studied. However, our systematic review also presents some differences: (1) we include exercise interventions plus supplementation (4 RCTs), (2) we only included RCTs with a detailed description of exercise, diet and supplementation interventions, (3) we included a larger number of variables (our review includes 51 variables, while the other reviews mentioned above include 8 variables [39], 20 variables [40] and 24 variables [41]).

We would like to clarify that there are 2 interventions [27,37] in which the raw data were not included. Two authors, T.P.B and A.F.S.J. made two attempts to contact the corresponding authors using the available e-mail addresses without response. Because of this, when discussing the results of the interventions and calculating the percentages of change these two interventions were not considered.

### 4.1. Anthropometry, Body Composition and Metabolic Biomarkers

Of the 12 interventions, improvements in anthropometry and body composition were found in 6 of 7 (85.7%) [27,28,31,33,35,36]. Our findings show that the combined interventions of diet plus exercise produced an average improvement of 9.9% (5.2–14.5%) in BMI, 1.7% (1.5–3.7%) in BM, 2.6% (1.6–3.6%) in waist circumference, 1.3% (1.1–1.5%) in hip circumference and 5.2% (3.2–7.1%) in FM. Increased BM and FM as well as decreased muscle mass in breast cancer patients has been associated with increased mortality, increased recurrence, decreased QoL and increased tumour-promoting sex hormones [42,43,44,45]. Maintenance of muscle mass is associated with decreased toxicity from chemotherapy and/or radiotherapy and increased anti-inflammatory myokines (e.g., IL-6), and suppresses tumour growth [46]. Several studies with combined diet plus exercise interventions in healthy [47] and breast cancer patients [48,49,50] have shown improvements in BM and FFM. Therefore, in line with the results of the present systematic review, a combined diet plus exercise intervention seems to be a good option to achieve anthropometric and body composition improvements.

The studies that had follow-up after the end of the intervention showed that the gains achieved are not maintained in some cases. In one intervention, after the end of the intervention and after follow-up, 38.7% of the gains in BM, 13.6% in waist circumference and 29.5% in hip circumference were lost [35]. However, in another intervention, the results were the opposite [28]. The results remained without significant change in the following variables, BM improved by 0.1% in the home group and by 0.7% in the community group, hip circumference worsened in the home group by 0.1% and by 0.2% in the community group, waist circumference worsened by 0.2% in the home group and improved by 0.5% in the community group and FM remained at identical values in the home and community groups [28].

One study found no improvement in any variable related to anthropometry and body composition [26]. A possible explanation for not finding any improvement could be that the EG experienced a reduction in total physical activity time (372.29 MET.min/wk) at the end of the intervention but hardly any change in energy intake (−11.73 Kcal). Finally, another intervention reported that FFM worsened at the end of the intervention, although the decline was not significant and worsened significantly after follow-up [28]. A possible explanation could be the fact that this intervention did not include strength work, which has shown improvements in FFM in cancer patients [51].

In the intervention that had to be withdrawn because it did not include the p-values, the EG decreased body mass by 9.4% during the intervention [38].

Of the 12 interventions, 4 of the 8 interventions (50.0%) showed improvement in metabolic biomarkers [28,29,34,36]. The combined interventions of diet plus exercise showed at the end of the interventions an average improvement in TC of 3.3% (3.1–3.5%) in the community group [28] and leptin improved by 7.5% [36]. In an intervention involving 4 groups (control, calcitriol, exercise and a combination of exercise plus calcitriol), a significant improvement in bone formation measured by Bone-specific Alkaline Phosphatase of 34.7% was observed in the group of participants taking calcitriol [29]. It is worth mentioning that this study analysed the data grouping all participants who exercised and those who did not and they also analysed the results grouping all participants who consumed calcitriol with those who did not. It did not analyse the results of the 4 groups with each other [29]. In another intervention, it was found that morning cortisol levels increased significantly (raw data not available) [34].

Regarding metabolic biomarkers, hyperinsulinemia and insulin resistance, as well as increased markers of low-grade inflammation such as IL-6, TNF-α and CRP, together with decreased anti-inflammatory factors such as adiponectin, elevated levels of oestrogens and androgens (i.e., estrone, oestradiol and testosterone) and low levels of sex hormone-binding globulin have been associated with breast cancer risk [52]. High levels of leptin have been associated with an increased risk of breast cancer as well as with a worse prognosis [53]; however, low levels of ghrelin are associated with increased expression of aromatase in adipose tissue and thus with an increased risk of breast cancer [54]. Increased lipids and lipoproteins contribute to the development of cardiovascular disease (CVD) [55,56]. Elevated cortisol levels in the morning and a decrease throughout the day represent the normal response [57], whereas elevated levels in the evening are associated with risk of depression [58]. Higher leukocyte levels appear to be associated with an increased risk of recurrence [59]. BMD decreases in BC patients treated with TC and/or HT, aromatase inhibitors for postmenopausal women and Tamoxifen in premenopausal women. These treatments cause a decrease in oestrogen production, resulting in bone loss [60]. Calcium and vitamin D are important nutrients for bone health [61]. Numerous studies have shown the benefits of combined interventions of exercise plus diet on lipid profile, insulin, adiponectin, leptin and ghrelin [50,54,62,63,64] in breast cancer patients. Combined interventions of exercise plus diet have also found benefits in markers of inflammation, oestrogens, androgens and SHBP in other patient populations [65]. Exercise program alone in breast cancer patients also found improvements in IGF-1, IGFBP-1 and IGFBP-3 [66] in maintenance of BMD [67].

In one study that had follow-up after the end of the intervention, the results remained unchanged. TC, LDL-C, TG, HOMA-IR improved by 0.2%, 2.6%, 0.6% and 2.3%, respectively, in the community group and systolic blood pressure and insulin worsened by 0.2% and 0.04%, respectively, in community group [28].

In the 4 interventions that did not find significant improvements, the reasons could be the low sample size [33,35], low adherence to exercise and/or diet/supplementation interventions [33,35,37], dietary intervention not tightly controlled [33], food questionnaires that did not adequately capture caloric intake [35] and exercise intensity was low enough to improve BMD [32], which would limit the ability to detect changes. In one intervention, although no significant improvements were detected, the EG lost less BMD than the CG [37].

### 4.2. Physical Function

Of the 12 interventions, physical function was analysed in 9 of them [26,27,28,29,32,33,35,36,37] and improvements were found in 5 interventions (55.6%) [26,27,28,36,37]. Our findings show that the combined interventions of exercise plus diet produce improvements of 4.4% in muscular endurance [26,27], 8.2% in the community group in 12MWT [28], 3.2% and 1.8% in systolic blood pressure in the community and home groups, respectively [28], 6.7% in diastolic blood pressure [36] and 32.2% in VO_2max_ [36]. Strength levels improved by an average of 22.9% in hip, knee and wrist flexion and extension [37]. In the study conducted by Arikawa et al. [38], EG improved cardiorespiratory fitness, as measured via a submaximal treadmill test, by 29.4%. The 12MWT improved by a further 2.2% during the 6-month follow-up period [28]. Elevated muscular strength and CRF levels represent a strong predictor of decreased mortality in breast cancer patients [68,69] and, in addition, high blood pressure contributes to the development of CVD [70]. Studies in breast cancer patients using combined interventions of exercise plus diet, exercise plus supplementation or exercise alone produced improvements in blood pressure [71], cardiorespiratory markers [72,73,74] and muscular strength [73,74,75]. Consequently, programs that combine exercise plus diet/supplementation seem to be a good option to improve physical function.

In 4 interventions [29,32,33,35], no significant improvements in physical function were observed, which may be due to displeasure with the measuring device (face masks) that could have caused them to terminate cardiorespiratory tests prematurely [33], less inclination to tolerate maximum discomfort in cardiorespiratory tests [33], low adherence to the exercise program [29,32,35] or low exercise intensity [32,33].

### 4.3. Healthy Lifestyles

Of the 12 interventions, healthy lifestyles were analysed in 8 interventions [26,27,28,30,31,32,33,35,36] and improvements were found in all of them (100%).

In relation to physical activity levels, in the only intervention that combined exercise plus supplementation, both groups improved activity levels and, although no significant differences were found between the two groups after the end of the intervention, physical activity levels increased by 11.3% and 8.3% in EG and CG, respectively [32]. Two interventions found an increase in time spent in moderate recreational activity or leisure time [26,27]. Another intervention found a 13.4% decrease in “somewhat” inactive time and a 49.5% increase in “somewhat” active time [33]. On the other hand, another intervention found a 133.3% improvement in the sport/exercise index [35]. Two interventions had follow-up periods [28,35]. In one of the interventions, at 12 months, the community group obtained significantly greater increases (43.0%) than the control and home groups and, in addition, was the only group that continued to improve its physical activity levels compared to baseline values [28]. The remaining intervention had a follow-up period of 6 months, resulting in a 48.6% loss of the gains obtained at the end of the intervention; however, levels were still significantly better than at baseline [35].

In the case of dietary intake, one intervention found an increase in fibre intake and a decrease in alcohol and animal protein intake [26]. Regarding total fat intake, two interventions found that a combined exercise and diet program produced a mean decrease of 14.0% (10.4–16.8%) [28,36] and regarding saturated fat intake, a mean reduction of 16.9% (14.2–20.0%) was found [28,35]. Total intake showed a significant mean reduction of 16.2% (11.1–21.3%). Two interventions were followed up on and resulted in a 15.0% increase in fibre intake and a 32.0% increase in fruit/vegetable intake 6 months after completion of the intervention [35]. The other intervention found that 12 months after starting the intervention, total fat intake and saturated fat intake remained unchanged from the end of the intervention in the home group, with decreases of 0.4% and 0.5%, respectively. However, the community group showed additional decreases of 3.8% in total fat intake and 2.5% in saturated fat intake [28].

Finally, only one intervention [31] analysed sleep quality and improved significantly in the EG (−75.6%). It is worth noting that the tool used to analyse sleep quality was the Pittsburgh Sleep Quality Index, where lower scores denote a healthier sleep quality.

These results are important because breast cancer patients are more likely to gain weight during chemotherapy [76] and obesity negatively affects the survival and prognosis of these patients [77]. In this sense, the case-control EpiGEICAM study showed that the adherence to the Western dietary pattern is related to a higher risk of BC (odds ratio (OR) for the top versus (vs.) the bottom quartile 1.46 (95% CI 1.06–2.01)), mainly in premenopausal women, whereas the Mediterranean dietary pattern was related to a lower risk (OR for the top quartile vs. the bottom quartile 0.56 (95% CI 0.40–0.79)), particularly of triple negative tumors and with no differences between pre- and postmenopausal women [78]. Further, one year after surgery, pre-operative physical activity levels were not recovered [79,80,81]. However, this systematic review and the literature has shown how physical activity and dietary interventions might facilitate weight loss [49,82].

### 4.4. Quality of Life

Of the 12 interventions, QoL was analysed in 6 interventions [26,27,28,30,31,33,36] and improvements were found in 5 of them (83.3%) [27,28,30,31,33,36]. At the end of the intervention period, improvements in different dimensions of the EORTC QLQ-C30 Questionnaire such as Global QoL, Physical Function, Role Function, Emotional Function and Cognitive Function were found [27]. In this same EORTC QLQ-C30 Questionnaire, improvements were found in Global QoL, Physical Function, Role Function, Emotional Function, Cognitive Function and Social Function which on average were 42.7% (29.4–58.7%) and an average reduction of 90.3% in breast cancer-related symptoms such as fatigue, nausea and vomiting, pain, dyspnea, insomnia, loss of appetite, constipation, diarrhea and financial difficulties [30]. Three interventions used the FACT-B Questionnaire and found improvements that on average were 15.1% (9.5–22.6%) [28,33,36]. Finally, an intervention using the EORTC QLQ-BR23 Questionnaire found improvements in the Body Image and Future Outlook subscales improved by 38.2% and 158.3%, respectively [31]. The side effects resulting from breast cancer treatment that patients may suffer could decrease their QoL levels [83]. Exercise ameliorates the side effects of cancer treatment such as sarcopenia, bone loss and cardiovascular disease [84]. Moreover, patients who were able to complete the exercise interventions experienced a sense of achievement and increased self-esteem [85]. All these reported results would positively influence the improvement of QoL. Furthermore, higher quality diets, such as the Mediterranean diet, have a positive impact on the QoL of breast cancer patients, specifically on physical functioning, sleep, pain and general well-being [86]. Weight gain and obesity negatively affect QoL [87], interventions with diet and/or exercise could reverse these negative outcomes [88].

In two interventions where follow-ups were conducted, 2.9% increases in FACT-B were found over the end of the intervention [28] and improvements were found in Global QoL at 6-month follow-up [27] and in Global QoL, Physical Function, Role Function and Social Function at 12 months [27].

In one intervention, no significant differences in QoL were found between the two groups studied [26]. A possible explanation could be that the patients started from similar or even higher baseline values than the general population and therefore finding improvements in QoL would be difficult. For the Global QoL domain, healthy women from the general population obtained a score of 66, as shown by normative data for the EORTC QLQ-C30 health-related quality of life questionnaire based on more than 15,000 participants from 13 European countries, Canada and the United States [89], while the patients’ initial score was 68.45 and 69.94 for the EG and CG, respectively [26].

The Sexual Function and Sexual Enjoyment subscales decreased in one of the interventions [31]. Sexual problems are highly prevalent in patients with breast cancer [90]. These sexual problems have been associated with the use of aromatase inhibitors [91] and tamoxifen [92]. Moreover, the surgical treatment, chemotherapy and hormone therapy appear to accelerate the onset of menopause and negatively influence sexual function [93].

### 4.5. Psychosocial Variables

Of the 12 interventions, psychosocial variables were analysed in 4 interventions and improvements were found in all (100%) [26,27,28,34]. The depression and anxiety values improved in all the interventions where they were analysed [26,27,34]. In the only intervention that presented raw data, the improvement in depression was 54.9% while the improvement in anxiety was 25.8% [34]. The other interventions [26,27] showed improvements in depression and anxiety but did not present the raw data. However, a noteworthy aspect was that at 6-month follow-up, the number of patients with a depression score >10 was lower in the EG (54.0%) than in the CG (69.9%) [26]. Regarding economic cost, the home and community groups showed lower costs than the control group, 9.5% and 7.4%, respectively [28].

Breast cancer patients are at increased risk for depression and anxiety [94] and stress [95]. Cancer is a major economic burden on society. Breast cancer alone accounts for 12% of the total expenditure on cancer treatment in the European Union (€126 billion in 2009) [96]. The results observed are in line with exercise interventions which have been shown to improve depression and anxiety in breast cancer patients [97,98]. On the other hand, a correlation has been found between elevated levels of depression and unhealthy diets [99] as well as an increased incidence of depression in overweight or obese breast cancer survivors with low levels of physical activity [100]. Then, the combined interventions of exercise plus diet could have positive effects on psychosocial variables in breast cancer patients.

### 4.6. Fatigue

Of the 12 interventions, fatigue was studied in 4 of them [25,26,27,31] and improvements were found in 2 (50%) [27,31]. At the end of the interventions, the combined exercise plus diet programs produced improvements in different dimensions of fatigue such as physical fatigue, mental fatigue, reduced activities and reduced motivation [27]. Another intervention produced a 64.5% improvement in CFS [31]. In this sense, it is important to highlight that fatigue is a side effect with a high prevalence (27%) in breast cancer patients [101]. Improvements in strength and cardiorespiratory fitness increase functional capacity and muscle mass and decrease fatigue levels [102]. In the same vein, some supplements such as CoQ10 are related to the generation of energy needed to preserve physical function and overall health, improving fatigue in cancer patients [103]. Dietary interventions may help to decrease chronic inflammation by decreasing inflammatory biomarkers such as CRP, IL6 [104] that are associated with fatigue in cancer patients [105].

In one study, no improvement was found after the end of the intervention or in the follow-up period [26]. However, in the follow-up period the EG experienced a smaller increase in fatigue (15%) compared to the CG (20%) [26]. In the other study, both groups experienced a substantial reduction in perceived fatigue; however, no significant differences were found between the two groups. The EG reduced general fatigue and physical fatigue by 28.4% and 32.2%, respectively, while the CG reduced general fatigue and physical fatigue by 20.1% and 32.1%, respectively [25]. In this case, antioxidant supplementation does not add benefit to strength training [25]. Possible causes could be that in both studies, the initial fatigue levels were already low, which could explain the results.

## 5. Limitations

The limitations of this systematic review are as follows: (1) the small sample size of the groups (8 of 12 interventions including <100 participants); (2) the heterogeneity of the exercise interventions (e.g., different types, frequency, intensity, type of session, volume of session, type of supervision); (3) the lack or short duration of follow-ups in most interventions; (4) the adherence to exercise in most of the interventions (6 of 12 interventions) was low (<80%); (5) some studies do not present raw data, making it difficult to interpret and present the data [27,37].

## 6. Conclusions

The results of this systematic review suggest that interventions combining exercise plus diet show significant improvements in CRF, muscular strength, body composition, QoL, fatigue, anxiety, depression and sleep. According to our analysis, combined exercise plus supplementation programs do not provide greater improvements than programs that employ only exercise or supplementation. Most of the exercise interventions analysed were semi-supervised or supervised, multicomponent (mainly strength and cardiorespiratory exercise), 3 to five days a week, with a duration of 24 weeks, with a session duration of 30 min, and developed at patients’ homes and exercise or heath care settings. The most used diet was a hypocaloric diet with a macronutrient composition of 30–35% lipids, 50–55% carbohydrates and 10–15% proteins. The supplementation employed was mainly vitamins.

Future lines of research should focus on RCTs with larger sample size, greater homogeneity of exercise interventions, interventions with improved strategies to increase exercise adherence (≥80%) and long-term follow-up periods. 

## Figures and Tables

**Table 1:** Characteristics of studies with exercise plus diet intervention.

Study	Year of Publication	Study Design	N	Age (yr)	Cancer Stage	Treatment	Type of Exercise	Type of Diet	Duration	Setting	Supervision	Outcomes
Jacot et al. [26]	2020	T0: Baseline T1: End of CT T2: End of RT T3: 1 year after inclusion	360 (180 EG/180 CG)	52.66 ± 9.69 EG/52.35 ± 10.09 CG	T stage: Tis, T1-T4. N stage: NX, N0-N3	Post surgery and pre CT and RT	ST: 2–5sets x 6–12 reps, 1d/week. CRT: 30–45 min 50–75% HRR 1d/week (walking, cycling, swimming, dancing)	Normocaloric diet. Macronutrient composition: 30–35% lipids, 50–55% carbohydrates and 10–15% proteins	26 weeks	Home/Hospital	Unsupervised/Supervised	FA; A&D; QoL; ME; Amount of PA; WC; BM; BMI; DI; CT Completion Rates
Carayol et al. [27]	2019	T0: Baseline T1: Mid-intervention T2: End of Intervention T3: 6-month follow-up T4: 1-year follow-up	143 (72 EG/71 CG)	51.2 ± 10.9 EG/52.1 ± 9.3 CG	I-IIIc	Post surgery and pre CT and RT	ST: 2–5sets x 6–12reps, 1d/week. CRT: 30–45 min 50–75% HRR 2d/week (walking, cycling, swimming, dancing)	Normocaloric diet. Macronutrient composition: 30–35% lipids, 50–55% carbohydrates and 10–15% proteins	26 weeks	Home/Hospital	Unsupervised/Supervised	FA; A&D; QoL; MS; MP; ME; Amount of PA; FM; MM; BM; BMI; DI; CT Completion Rates
Harvie et al. [28]	2019	T0: Baseline T1: Change at 6 months T2: Change at 12 months	409 (138 CG/134 HBP/137 CBP)	55.3 ± 10.5 CG/54.6 ± 11.2 HBP/54.0 ± 9.2 CBP	Invasive or in-situ primary BC	Post surgery	HBP: Individualised PA advice (not described). CBP:Supervised sessions. CRT 30 min 50–80% HRM 12sessions/week. 10 min ST + flexibility 12sessions/week	Weight Maintenance diet or Hypocaloric diet (25% ED). Macronutrient composition: 30% lipids, 45% carbohydrates and 25% proteins	12 weeks	Home/Community locations	Unsupervised/Supervised	BM; WC; HC; BP; Insulin; Glucose; HOMA-IR; TC; LDL-C; HDL-C; TG; FM; FFM; 12MWT; Qol; DA; PAA; Patient-specific costs; Amount of PA
Ghavami and Akyolcu [30]; Ghavami and Akyolcu [31]	2017	T0: Baseline T1: Change at 24 weeks	80 (40 CG/40EG)	49.23 ± 9.46 CG/48.75 ± 9.49	I-III. ER/PR/HER2negative	Treatment completed (Surgery, CT, RT) ≥ 3 months	CRT: 30 min 70–85% HRR 3–5d/week (walking, stepping, cycling)	Diet goal: Reduction the total daily calorie intake to 600 kcal below calculated energy requirements	24 weeks	Exercise Facility and Home	Supervised	QoL; BMI; FA; Sleep Quality
Swisher et al. [33]	2015	T0: Baseline T1: Change at 12 weeks	28 (10 CG/18 EG)	53.8 EG/53.6 CG	I-III. ER/PR/HER2negative	Treatment completed > 3 months	CRT: 30 min 60–75% PHR (11–14 RPE) 5d/week	Diet goal: Decrease dietary fat caloric intake by 200 kcal per week	12 weeks	Home/Exercise Facility	Unsupervised/Supervised	BM; BMI; FM; WC; HC; VO2peak; QoL; IL-6; TNF-α; Adiponectin; Insulin; Leptin; Amount of PA
Saxton et al. [34]	2014	T0: Baseline T1: Change at 24 weeks	85 (41 CG/44EG)	55.8 ± 10.0 EG/55.3 ± 8.8 CG	I-III	Treatment completed (Surgery, CT, RT) 3–18 months	CRT: 30 min 65–85% HRM 3d/week (treadmill, cross- trainer, cycle ergometer, rowing ergometer) ST: 10–15 min, resistance bands, hand weights and stability balls	Diet goal: Reduction the total daily calorie intake to 600 kcal below calculated energy requirements	24 weeks	NR	Supervised	Depressive symptoms; Perceived Stress; IL-6; TNF-α; Leukocyte; Cortisol
Greenlee et al. [35]	2013	T0: Baseline T1: Change at 3 months T2: Change at 6 months T3: Change at 12 months	42 (22 EG/20 CG)	52.6 ± 8.0 EG/48.6 ± 9.6 CG	0-IIIa	Treatment completed > 6 months	Circuit Training: 30s ST/30s CRT. 1–7 wk: ≤60% HRM. ≥8 wk: 70–75% HRM, 3–5d/week (pneumatic resistance machine)	1–2 wk: 1200 cal/day, followed by 1600 cal/day Macronutrient composition: 25% lipids, 30% carbohydrayes and 45% proteins	6 months	Exercise Facility	NR	BM; WC, HC; FM; FFM; VO2max; TC; LDL-C; HDL-C; TG; Glucose; Insulin; Ghrelin; IGF-I; IGFBP-1; IGFBP-3; Adiponectin; CRP; HOMA-IR; DI; IA; Amount of PA
Scott et al. [36]	2013	T0: Baseline T1: Change at 24 weeks	90 (47 EG/43 CG)	55.6 ± 10.2 EG/55.9 ± 8.9 CG	I-III	Treatment completed (Surgery, CT, RT) 3–18 months	CRT: 30 min 65–85% HRM 3d/week (treadmill, cross- trainer, cycle ergometer, rowing ergometer) ST: 10–15 min, resistance bands, hand weights and stability balls	Diet goal: Reduction the total daily calorie intake to 600 kcal below calculated energy requirements	24 weeks	Exercise Facility	Supervised	BM; BMI; DI; WC; FM; BP; 8MWT; RHR; QoL; HOMA-IR; SHBG; TT; E1; E2; IGF-I; IGFBP-1; IGFBP-3; Leptin; CRP; TC; HDL-C

8MWT: 8 min walking test; 12MWT: 12 min treadmill walking test; A&D: Anxiety and Depression; ANC: Axillary node clearance; BC: Breast Cancer; BCS: Breast-conservating Surgery; BM: Body Mass; BMI: Body Mass Index; BP: Blood Pressure; CBP: Community-based Programme; CG: Control Group; CRP: C-Reactive Protein; CRT: cardiorespiratory Training; CT: Chemotherapy; DA: Dietary Adherence; DBP: Diastolic Blood Preassure; DI: Dietary Intake; E1: Estrone; E2: Estradiol; ED: Energy Deficit; EG: Experimental Group; ER: Estrogen Receptor; EX: Exercise; FA: Fatigue; FACT TOI: Functional Assessment of Cancer Therapy. Trial Outcome Indicator; FFM: Fat Free Mass; FM: Fat Mass; HAES: Habitual Activity Estimation Scale; HBP: Home-based Programme; HC: Hip Circumference; HDL-C: High-Density Lipoprotein Cholesterol; HER2: Human Epidermal Growth Factor Receptor 2; HOMA: Homeostatic Model Assessment of Insulin Resistance; HRM: Heart Rate Maximum; HRR: Heart Rate Reserve; IA: Intervention Adherence; IGF-I: Insulin-Like Growth factor-I; IL-6: Interleukin-6; IGFBP: Insulin-like Growth Factor Binding Protein; LDL-C: Low-Density Lipoprotein Cholesterol; Lx: Lumpectomy; ME: Muscle Endurance; MP: Muscle Power; MS: Muscle Strength; MT: Mastectomy; NR: Not Reported; NS: Not Significant; PA: Physical Activity; PAA: Physical Activity Adherence; PHR: Peak Heart Rate; PR: Progesterone Receptor; PSQI: Pittsburgh Sleep Quality Index; QoL: Quality of Life; QT: Quadrantectomy; RHR: Resting Heart Rate; RPE: Rating of Perceived Exertion; RT: Radiotherapy; SHBG: Sex Hormone-Binding Globulin; ST: Strength Training; TC: Total Cholesterol; TG: Triglycerides; TNF-α: Tumour Necrosis Factor alpha; TT: Testosterone; V02 = Oxygen uptake; WC: Waist Circumference; WHR: Waist/Hip ratio.

**Table 2:** Characteristics of studies with exercise plus supplementation intervention.

Study	Year of Publication	Study Design	N	Age (yr)	Cancer Stage	Treatment	Type of Exercise	Type of Supplementation	Duration	Setting	Supervision	Outcome
de Lima et al. [25]	2020	T0: Baseline T1: Change at 14 weeks	25 (12 EG/13 CG)	51.00 ± 9.03 EG/48.23 ± 8.34 CG	I-IIIc	Major treatments completed ≥ 6 months	ST: 4–8 wk (3sets × 10–12 RM), 9–13 wk (3sets × 8–10 RM). 2d/week. (Weight machines)	250 mg VIT C + 90 mg VIT E	10 wk ST, 12 wk VIT	N/R	Supervised	FA
Kim et al. [32]	2016	T0: Baseline T1: Change at 6 months	43 (23 EG/20 CG)	55.7 ± 5.3 EG/56.3 ± 6.7 CG	0-III	Primary treatment completed ≥ 3 months	CRT: 20–60 min RPE 11–13 2–5d/week (walking). ST:2sets × 8–10reps low-moderate intensity 2–3d/week (elastic bands)	500 mg Calcium + 1000 IU VIT D	6 mo	Home	Unsupervised	BMD, NTx, Serum calcium, Serum 25(OH)D, Amount of PA, MS, 6MWT
Peppone et al. [29]	2018	T0: Baseline T1: Change at 12 weeks	41 (10 Calcitriol/10 Exercise/11 Calcitriol + Exercise/10 CG)	53.5 ± 7.8	0-III	Five year window after diagnosis and receiving HT	CRT: 60–70% HRR 7d/week. Maximum 12,000 steps/day. RPE 3–5/10 (walking). ST: Elastic bands, RPE 5–8/10. 1–3sets × 7–10reps, 10 exercises (squats, side bends, leg extensions, leg curls, chest press, rows, toe raises, overhead press, biceps curls, triceps extensions), 3d/week	45 micrograms (mcg) of calcitriol once weekly.	12 wk	Home	Unsupervised	NTx, BSAP, BRI, MS, VO_2max_
Waltman et al. [37]	2010	T0: Baseline T1: Change at 12 months T2: Change at 24 months	223 (110 EG/113 CG)	58.69 ± 7.5	0-II	Completed breast cancer treatment (except tamoxifen and aromatase inhibitors) ≥ 6 months earlier	ST: 1–9 mo (30–45 min exercises with ≤20lb hand or ankle weights, 2d/week), 10–24 mo (8 exercises, 2sets × 8–12reps, 2d/week). Balance exercises	1200 mg Calcium and 400 IU VIT D daily and 35 mg risedronate weekly	24 mo	Home/Fitness Center	Unsupervised/Supervised	MS, Balance, BMD, Alkphase B, NTx

Serum 25(OH)D: Vitamin D; 6MWT: 6 min Walking Test; BMD: Bone Mineral Density; BRI: Bone Remodeling Index; BSAP: Bone-specific Alkaline Phosphatase; CG: Control Group; CRT: cardiorespiratory Training; EG: Experimental Group; FA: Fatigue; HT: Hormone Therapy; IU: International Units; MS: Muscle Strength; NTx: Type I Collagen linked N-telopeptide; PA: Physical Activity; RM: Repetition Maximum; RPE: Rating of Perceived Exertion; ST: Strength Training; VIT: Vitamin; VO_2_: Oxygen uptake.

**Table 3:** Methodological quality of included studies.

Items												
Study	1	2	3	4	5	6	7	8	9	10	11	Total Score (0–10)
de Lima et al. [25]	1	1	0	1	1	1	1	1	1	1	1	9
Jacot et al. [26]	1	1	0	1	0	0	0	1	1	1	1	6
Carayol et al. [27]	1	1	1	1	0	0	0	1	1	1	1	7
Harvie et al. [28]	1	1	0	1	0	0	0	1	1	1	1	6
Peppone et al. [29]	1	1	0	1	0	0	0	1	1	1	1	6
Ghavami and Akyolcu [30]; Ghavami and Akyolcu [31]	1	1	0	1	0	0	0	1	1	1	1	6
Kim et al. [32]	1	1	1	1	1	0	1	1	1	1	1	9
Swisher et al. [33]	1	1	1	1	0	0	0	1	1	1	1	7
Saxton et al. [34]	1	1	1	1	0	0	1	1	1	1	1	8
Greenlee et al. [35]	1	1	0	1	0	0	0	1	1	1	1	6
Scott et al. [36]	1	1	1	1	0	0	1	1	1	1	1	8
Waltman et al. [37]	1	1	0	1	0	0	0	1	1	1	1	6

PEDro items: 1 Eligibility criteria; 2 Random allocation; 3 Concealed allocation; 4 Comparability at baseline; 5 Patient blinding; 6 Therapist blinding; 7 Assessor blinding; 8 At least 85% follow-up; 9 Intention to treat analysis; 10 Between-group statistical comparisons; 11 Point measures and measures of variability.

**Table 4:** Results of studies with exercise plus diet intervention.

Study	Compliance Rate	Anthropometry, Body Composition and Metabolic Biomarkers	Physical Function	Healthy Lifestyles	QoL	Psychosocial Variables	Fatigue
Jacot et al. [26]	47.2% exercise	No changes within or between-groups	T1(EGvsCG): - ME (repetitions) ↑	T1(EGvsCG): - Recreational Moderate Intensity (MET.min·wk^−1^) ↑ T2(EGvsCG): - Recreational Moderate Intensity (MET.min·wk^−1^) ↑ - Fiber (g) ↑ - Animal Proteins (g) ↓ - Alcohol (g) ↓ T3(EGvsCG): - Total MET (MET.min·wk^−1^) ↑	No significant differences between groups	T3(EGvsCG): - Depression score > 10 (patients) ↓	No significant differences between groups
Carayol et al. [27]	97% nutrition; 67% exercise	T2(EGvsCG): - BMI (kg/m^2^) ↓ - FM (%) ↓ - FM-trunk(kg) ↓ T3(EGvsCG): - MM-legs(kg) ↑	T2(EGvsCG): - ME (repetitions) ↑	T1(EGvsCG): - Leisure time (MET.min/wk^−1^) ↑ T2(EGvsCG): - Leisure time (MET.min/wk^−1^) ↑	T1(EGvsCG): - Global QoL (points) ↑ T2(EGvsCG): - Global QoL (points) ↑ T3(EGvsCG): - Global QoL (points) ↑ T4(ECvsCG): - Global QoL (points) ↑	T1(EGvsCG): - Anxiety(points) ↓ - Depression (points) ↓ T2(EGvsCG): - Anxiety(points) ↓ - Depression (points) ↓	T1(EGvsCG): - Physical Fatigue (points) ↓ - Mental Fatigue (points) ↓ - Reduced activities ↓ - Reduced Motivations ↓ T4(EGvsCT): - General Fatigue (points)↓ - Physical Fatigue (points)↓ - Reduced activities ↓
Harvie et al. [28]	CBP 64% exercise and nutrition	T1(HBPvsCG)/(CBPvsCG): - BM (kg) ↓/↓ - FM (kg) ↓/↓ - WC (cm) ↓/↓ - HC (cm) ↓/↓ - FM-trunk (kg) ↓/↓ - TC (mmol/L)NS/↓ - LDL-C (mmol/L)NS/↓ T2(HBPvsCG)/(CBPvsCG): - BM (kg) ↓/↓ - FM (kg) ↓/↓ - FFM (kg) ↓/ - WC (cm) ↓/↓ - HC (cm) ↓/↓ - FM-trunk (kg) ↓/↓ - TC (mmol/L) NS/↓ - LDL-C (mmol/L) NS/↓ - TG (mmol/L) NS/↑ - Insulin (pmol/L) NS/↑ - HOMA NS/↑	T1(HBPvsCG)/(CBPvsCG): - 12MWT (m) ↑/↑ - SBP (mmHg) ↓/↓ T2(HBPvsCG)/(CBPvsCG): - SBP (mmHg) NS/↓	T1(HBPvsCG)/(CBPvsCG): - Energy Intake (Kcal) ↓/NS - Total Fat (g) ↓/↓ - Saturated Fat (g) ↓/↓ T2(CBPvsCG)/(CBPvsHBP): - Total moderate and vigorous physical activity (minutes/week) ↑/↑ - Energy Intake (Kcal) ↓/↓ - Total Fat (g) ↓/↓ - Saturated Fat (g) ↓/↓ - Carbohydrate (g) ↓/NS	T1(CBPvsCG): - FACT TOI-BC (points) ↑ T2(CBPvsCG): - FACT TOI-BC (points) ↑	HBP: £7737 CBP: £7914 CG: £8547	Not measured
Ghavami and Akyolcu [30]; Ghavami and Akyolcu [31]	NR	[30] T1(EG): - BMI (kg/m^2^) ↓	Not measured	[30] T1(EG): - PSQI (points) ↓	[29] T1(EG): - Global Health Status QoL (points) ↑ - Cancer-related Symptoms (points) ↓ [30] T1(EG): - Body image(points) ↑ - Sexual functioning (points) ↓ - Sexual enjoyment (points) ↓ - Future perspective (points) ↑ - Cancer-related Symptoms (points) ↓	Not measured	[30] T1(EG): - CFS (points) ↓
Swisher et al. [33]	≥80% nutrition and exercise	T1(EG): - BMI (kg/m2) ↓ - FM (%) ↓ - BM (kg) ↓	No changes within or between-groups	T1(EG): - Exercise time (min) ↑ - HAES somewhat inactive (min) ↓ - HAES somewhat active (min) ↑	T1(EG): - FACT B physical well-being (points)↑ - FACT B emotional well-being (points) ↑ - FACT B breast cancer-specific items (points) ↑ - FACT B Total (points) ↑	Not measured	Not measured
Saxton et al. [34]	84% exercise;	T1(EG): - Diurnal Cortisol (μg/dL) ↑	Not measured	Not measured	Not measured	T1(EG): - Depression(points) ↓ - Stress(points) ↓	Not measured
Greenlee et al. [35]	>80% nutrition; 36.7% exercise	T1(EG/CG): - BM (kg) ↓/NS - WC (cm) ↓/NS - HC (cm) ↓/NS T2(EG/CG): - BM (kg) ↓/↓ - WC (cm) ↓/NS - FM (%) ↓/(NS) - FM (kg) ↓/(NS)	No significant differences between groups	T2(EG/CG): - Sports/exercise index ↑/NS - Energy Intake (Kcal) ↓/↓ - Percent Protein (%) ↑/NS T3(EG/CG): - Sports/exercise index ↑/↑ - Energy Intake (Kcal) NS/↓ - Fruits &Vegetables(servings) ↑/NS - Fiber (g) ↑/(NS)	Not measured	Not measured	Not measured
Scott et al. [36]	80% nutrition and execise sessions	T1(EGvsCG): - WC (cm) ↓ - WHR ↓ - Leptin (pg/mL) ↓ - TC (mmol/L) ↓	T1(EGvsCG): - VO_2max_ (ml·kg^−1^·min^−1^) ↑ - DBP (mmHg) ↓	T1(EGvsCG): - Total Fat (g) ↓ - Saturated fat (g) ↓	T1(EGvsCG): - FACT B (points) ↑ - Cancer subscale (points) ↑	Not measured	Not measured

↑: Increase; ↓: Decrease; 12MWT: 12 min treadmill walking test; BM: Body Mass; BMI: Body Mass Index; CBP: Community-based Programme; CG: Control Group; DBP: Diastolic Blood Preassure; EG: Experimental Group; FACT TOI: Functional Assessment of Cancer Therapy. Trial Outcome Indicator; FFM: Fat Free Mass; FM: Fat Mass; HAES: Habitual Activity Estimation Scale; HBP: Home-based Programme; HC: Hip Circumference; HDL-C: High-Density Lipoprotein Cholesterol; ME: Muscle Endurance; NS: Not Significant; PSQI: Pittsburgh Sleep Quality Index; QoL: Quality of Life; TC: Total Cholesterol; V02 = Oxygen uptake; WC: Waist Circumference; WHR: Waist/Hip ratio.

**Table 5:** Results of studies with exercise plus supplementation intervention.

Study	Supplementation/Exercise Compliance Rate	Anthropometry, Body Composition and Metabolic Biomarkers	Physical Function	Healthy Lifestyle	Fatigue
de Lima et al. [25]	100% exercise and supplementation	Not measured	Not measured	Not measured	No significant differences between groups
Kim et al. [32]	69.5% for weight-bearing exercise, 48.5% for resistance exercise. Supplement adherence in EG was 84.3%	No significant differences between groups	No significant differences between groups	No significant differences between groups	Not measured
Peppone et al. [29]	89.5% took 100% of calcitrol pills; 10.5% took 83.3% of pills. Exercise NR	↑ BSAP Calcitriol group	No significant differences between groups	Not measured	Not measured
Waltman et al. [37]	69.4% exercise. 96.2% Risedronate. 93.7% Calcium/vitamin D	No significant differences between groups	MS ↑ although it is not indicated whether the improvement is statistically significant or not	Not measured	Not measured

↑: Increase; BSAP: Bone-specific Alkaline Phosphatase; EG: Experimental Group; NR: Not Reported.

**Table 6:** Summary of the improvements found in the outcome variables by intervention.

Study	CRF	Muscular Strength	Body Composition	PA Levels/Dietary Intake	QoL	Fatigue	Anxiety	Depression	Sleep
de Lima et al. [25]									
Jacot et al. [26]		×		×			×	×	
Carayol et al. [27]		×	×	×	×	×	×	×	
Harvie et al. [28]	×		×	×	×				
Peppone et al. [29]									
Ghavami and Akyolcu [30]; Ghavami and Akyolcu [31]			×	×	×	×			×
Kim et al. [32]				×					
Swisher et al. [33]			×	×	×				
Saxton et al. [34]							×	×	
Greenlee et al. [35]			×	×					
Scott et al. [36]	×		×	×	×				
Waltman et al. [37]		×							

CRF: Cardiorespiratory fitness; PA: Physical activity; QoL: Quality of life.

## Data Availability

Not applicable.
